# Realization of circular polarized multiple band multi-mode OAM antenna using a ring patch for IoT applications

**DOI:** 10.1038/s41598-023-43836-x

**Published:** 2023-10-10

**Authors:** Umar Fayyaz, Shahab Ahmad Niazi, Khaled AlJaloud, Abdul Aziz, Waqar Ahmad Malik, Rifaqat Hussain

**Affiliations:** 1 Faculty of Engineering and Technology, The Islamia University of Bahawalpur, Punjab, 63100 Pakistan; 2 College of Engineering, Muzahimiyah Branch, King Saud University, P.O. Box 2454, Riyadh, Saudi Arabia; 3 Department of Avionics Engineering, College of Aeronautical Engineering(CAE), National University of Sciences and Technology (NUST), Risalpur, Khyber-Pakhtunkhwa Pakistan; 4 Antenna and Electromagnetics Research Group, School of Electronic Engineering and Computer Science, Queen Marry University of London, London, UK

**Keywords:** Engineering, Electrical and electronic engineering

## Abstract

A multiband and multi-mode antenna with circular polarized conical patterns is suitable for achieving desired spectral efficiency, increased capacity, and spatial diversity for IoT applications. However, simultaneous excitation of such circular polarized multiple Orbital Angular Momentum (OAM) modes through a single patch antenna is challenging due to the complexity of simultaneously fulfilling distinct requirements of each mode. In this paper, a ring patch antenna is designed to excite different OAM states at different frequencies simultaneously. First, characteristic mode analysis is used to analyze the possibility of simultaneous excitation of multiple OAM modes at corresponding frequencies through a simple ring patch antenna. Then, a dual port ring patch antenna is designed and fabricated to verify the capability of generating multiple OAM states at corresponding frequencies. Furthermore, it also presents the guidance to suppress unwanted OAM modes.

## Introduction

The demand for explosive data traffic has increased tremendously due to rapid expansion in the Internet of things (IoT)^1^. However, it led to resource allocation and network congestion issues, thereby introducing challenges for communication technologies. 5G radio networks are used to support three different device connectivities, ultra-reliable low latency communication (URLLC), massive machine type communication (mMTC) and extended mobile broadband (eMBB)^2^. Particularly, eMBB is crucial for smart sensing devices in new era of IoT to increase he network spectral efficiency and user data rates, such as virtual reality (VR) and ultra-high quality video streaming^3–5^. While URLLC and mMTC enable IoT connectivity for machine-type traffic^6^.

EM waves with OAM have been reported as a promising solution to increase spectrum utilization efficiency for IoT applications^7^. The traditional approaches to increase the channel capacity have been extensively explored in the last few decades such as time and frequency division multiplexing^8^. Consequently, enhancing channel capacity or supporting more users using these resources becomes more challenging. However, 5G and beyond 5G future networks are expected to be developed using new modes domain through the orthogonal access of multiple users since the introduction of orbital angular momentum (OAM) for radio domain in 2007^9^. Theoretically, an electromagnetic wave may carry infinite orthogonal OAM states. So, simultaneous excitation of multiple OAM modes may help to achieve the desired increased data capacity for future wireless communication applications^10–14^.

Several techniques have been proposed to generate RF or mm-wave carrying OAM^15–29^. Intelligent metasurfaces^30^ and chiarilty assited phase metasurfaces^31^ have been used for advanced control of electromagnetic waves and effective decoupling of circular polarized components, respectively. Among these approaches, spiral phase plate^32^ has the simplest structure, which has been used widely for the generation of OAM beam. However, it can generate only one OAM mode once the design is decided. Furthermore, antenna array^33^ is another widely used approach for generating multiple OAM modes by controlling phase between adjacent elements. However, the complexity and cost of the system is increased with the increase of OAM modes.

Single patch antenna to excite circular polarized OAM waves are proposed in^27,34,35^. However, these could excite only one mode at a time. Furthermore, several single patch antennas have been proposed in^36–40^ to excite multiple conical patterns of $TM_{nm}$ modes using complex structure of substrate integrated waveguides (SIW), however excited modes are not circularly polarized. A multimode concentric ring patch antenna is proposed in^41^. However, it has a separate ring and dual port configuration for the excitation of each mode.

This work presents the design of a dual port single ring patch antenna to excite multiple OAM modes at different frequencies. It can generate multiple circular polarized OAM states at different frequencies simultaneously and suppress undesired states effectively. Each excited OAM mode (*l* = 2, 4 and 6) is associated with corresponding $TM_{n1}$ mode ($TM_{31}$, $TM_{51}$ and $TM_{71}$), respectively, at their corresponding frequencies. With the increase of mode number, the divergence angle and radius of the vortex OAM wave also increases. So, OAM waves with different mode numbers can cover different users or IoT devices around the antenna along the smaller, medium, and larger radial distances of the corresponding OAM mode^42^. The multi-band feature of the proposed work validates its feasibility for high-speed IoT devices for W-LAN, Wi-MAX, and 5G applications^2,43,44^. The transmission of multiple data streams using different OAM modes at their corresponding frequencies increases the capacity of IoT networks without affecting conventional communication wireless networks spectrum. Therefore, the proposed antenna is suitable for such applications where many IoT devices are required to communicate in a crowded frequency spectrum. Furthermore, the proposed method can be used for wireless backhaul in cellular devices, thereby increasing the capacity and reducing latency between base stations.

This paper is organized as follows. Section II of this paper discusses the modal analysis and configuration of the proposed antenna. Simulated results for the proposed antenna are discussed in Section III, while the measured performance of the fabricated prototype and its comparison with previous works are presented in Section IV. Finally, in Section V, the conclusion of the proposed work is presented.

## Analysis and design consideration

### Theoretical background

The basic idea to excite multiple OAM modes at multiple corresponding frequencies through a simple ring patch is conceived from the relation for the resonant frequency of a circular patch antenna for a corresponding $T_{n1}$ mode is given as follows^45,46^:

1 $$\begin{aligned} f_{n1}=\frac{X_{n1}C}{2\pi a_{e}\sqrt{\varepsilon _{r}}} \end{aligned}$$

Where *C* is the speed of light and $X_{n1}$ is the n-th zero of the derivative of Bessel function of order *n* for different modes^47^, $a_e$ is the effective radius of the circular patch antenna. The terms *n* and *m* represent the angular and radial modes. In this relation, it can be seen that the term $X_{n1}$ is directly proportional to $f_{n1}$ while keeping the radius $a_e$ constant. Based on this direct relation, a circular patch antenna configuration may help to excite multiple $T_{n1}$ modes at different frequencies for the fixed value of radius, theoretically.

The radiated electric field components excited by a $TM_{n1}$ mode are as follows^46^:

2 $$\begin{aligned} \begin{aligned} E_{\theta n} =&j^{n}\frac{Vk_{o}a}{2}\frac{e^{-jk_{o}r}}{r}cosn\phi [J_{n+1}(\gamma ) -J_{n-1}(\gamma ) ] \\E_{\phi n}=&j^{n}\frac{Vk_{o}a}{2}\frac{e^{-jk_{o}r}}{r} cosn\theta sin(n\phi ) [J_{n+1}(\gamma )+J_{n-1}(\gamma ) ] \end{aligned} \end{aligned}$$

In the above expression, $V=hE_{o}J_{n}(ka)$ represents the edge voltage, $E_{o}$ is the electric field at the patch edge, *h* is substrate thickness, *a* is patch radius and $J_i$ is Bessel function of order *i*. The non-zero OAM beam can be generated from a circular polarized (CP) patch antenna as investigated in^27^.

The superposition of individual field components causes the excitation of a required circular polarized OAM mode as follows:

3 $$\begin{aligned} \begin{aligned} {E_{\theta n}}^t= {E_{\theta n}}^1(\phi ,\theta )+ j{E_{\theta n}}^2(\phi +\alpha ,\theta ) \\ {E_{\phi n}}^t={E_{\phi n}}^1(\phi ,\theta )+ j{E_{\phi n}}^2(\phi +\alpha ,\theta ) \end{aligned} \end{aligned}$$

The superscripts 1 and 2 in Eq. (3) represent the individual field components radiated by both feeds. Considering the phase shift as $\pi /2$ for the RHCP patch antenna, the above expression is further derived to obtain *x* and *y* components of the total electric field as follows:

4 $$\begin{aligned} \begin{aligned} E_{x}= -j^{n}\frac{e^{-jk_or}}{2r}ahk_oJ_n(ak_o\sqrt{\varepsilon _r})[e^{-j(n-1)\phi }J_{n-1}(\gamma ) - e^{-j(n+1)\phi }J_{n+1}(\gamma ) ] cos[\theta ] \\ E_{y}= j^{n+1}\frac{e^{-jk_or}}{2r}ahk_oJ_n(ak_o\sqrt{\varepsilon _r})[e^{-j(n-1)\phi }J_{n-1}(\gamma )+ e^{-j(n+1)\phi }J_{n+1}(\gamma ) ] cos[\theta ] \end{aligned} \end{aligned}$$

The electric field components can further be written as follows:

5 $$\begin{aligned} \begin{aligned} E_x=&Ae^{-j(n-1)\phi }-Be^{-j(n+1)\phi } \\ E_y=&-j[Ae^{-j(n-1)\phi }+Be^{-j(n+1)\phi }] \end{aligned} \end{aligned}$$

From Eqs. (4) and (5), A and B are written as:

6 $$\begin{aligned} \begin{aligned} A=&-j^{n}\frac{e^{-jk_or}}{2r}ahk_oJ_n(ak_o\sqrt{\varepsilon _r})cos[\theta ]J_{n-1}(\gamma ) \\ B=&-j^{n}\frac{e^{-jk_or}}{2r}ahk_oJ_n(ak_o\sqrt{\varepsilon _r})cos[\theta ]J_{n+1}(\gamma ) \end{aligned} \end{aligned}$$

The electric field components $E_x$ and $E_y$ in the above expression (5) have two same terms *A* and *B*, with their difference and sum, respectively. Furthermore, these terms contain phase factors of form $e^{-j(n-1)\phi }$ and $e^{-j(n+1)\phi }$ respectively. The amplitude of A and B correspond to the order of the Bessel function, and the term $e^{-j(n-1)\phi }$ is responsible for the excitation of RHCP OAM wave of order $n-1$ for $TM_{n1}$ mode. In a similar way, LHCP OAM wave of order $-(n-1)$ with dominant phase factor $e^{j(n-1)\phi }$, can also be excited by considering the phase shift as $-\pi /2$.

A specific circular polarized $TM_{n1}$ mode can be excited effectively through the excitation of two feed points separated by a corresponding angular spacing of ’$\alpha$’, which should be an odd multiple of 360/4*n* degrees^46^. Moreover, the two feed points should be excited simultaneously with excitation fields of the same amplitudes but a relative phase shift of $-\pi /2$^48^. Generally, an antenna radiating an EM wave of $TM_{n1}$ mode can generate OAM wave with a mode order of $\pm (l = n-1)$. Therefore, the antenna design can be done after determining the harmonic relationship between patch size, operating frequency and mode order. So, by adjusting the radius of a circular patch and angular spacing between the two feed points, an electromagnetic wave of a specific circular polarized OAM mode can be excited^41,47^. However, simultaneous excitation of such circular polarized multiple Orbital Angular Momentum (OAM) modes through a single patch antenna is challenging due to the complexity of simultaneously fulfilling distinct specific requirements of each mode.

**Figure 1:**
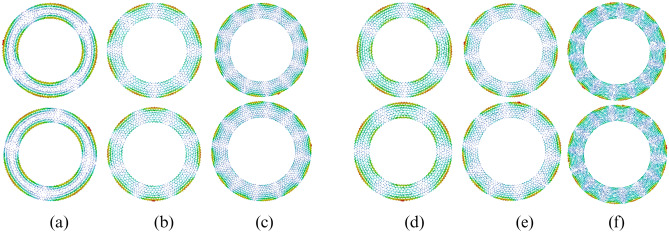
Characteristic current distribution on antenna surface (**a**) $l=1$ OAM mode ($\mathrm {TM_{21}}$) at 3.05 GHz (**b**) $l=3$ OAM mode ($\mathrm {TM_{41}}$) at 4.04 GHz (**c**) $l=5$ OAM mode ($\mathrm {TM_{61}}$) at 5.28 GHz (**d**) $l=2$ OAM mode ($\mathrm {TM_{31}}$) at 3.49 GHz (**e**) $l=4$ OAM mode ($\mathrm {TM_{51}}$) at 4.63 GHz (**f**) $l=6$ OAM mode ($\mathrm {TM_{71}}$) at 5.92 GHz.

### Characteristic mode analysis

Characteristic mode analysis (CMA) may help analyze the possibility for simultaneous excitation of multiple OAM modes at corresponding multiple frequencies through a single patch. For this purpose, a ring patch is selected due to its symmetrical shape, which is suitable for achieving circular polarization. The CMA is performed for the ring patch in CST Microwave Studio. The analysis is also helpful to realize proposed antenna in a controlled manner due to its physical insight to select possible excitation points^49–54^. According to mode synthesis theory, the excitation of a specific order OAM mode can be achieved by the excitation of two degenerate modes with 90° phase difference and specific angular separation between excitation points^35^.

Figure 1 represents the distribution of characteristic currents on the ring patch surface at six different frequencies. The characteristic current distributions are shown pairwise for each pair of possible degenerate modes, and such pairs of three odd modes and three even mode pairs are shown here. The OAM wave of a specific order can be obtained by simultaneous excitation of the corresponding pair of degenerate modes at each frequency. The characteristic currents in Fig. 1a,b,c can generate odd-order OAM waves of mode first, third and fifth, respectively at their corresponding frequencies. Similarly, even order OAM waves of mode second, fourth and sixth can be obtained at their corresponding frequencies as shown in Fig. 1d,e,f. Hence, the characteristic mode analysis of the ring patch structure verifies the possibility that six different OAM waves at corresponding multiple frequencies can be excited from a single ring patch of fixed radius, as demonstrated in Eq. (1).

## Realization of multiband and multimode ring patch antenna

The angular separation between the two feed points should be an odd multiple of 360/4*n* degrees to excite effective circular polarized $TM_{n1}$ modes^47^. Based on this theory, multiple OAM modes $(l = n-1)$ may have different angular separation between two excitation points, so one may need multiple excitation points to excite multiple circular polarized OAM modes. However, fortunately 90 degrees angular separation between two feed points is one of the odd multiples of 360/4*n* for all the three even order OAM modes $(l = 2, 4~ and~6)$. So, two ports with 90-degree separation may simultaneously excite these even-order circular polarized OAM modes if both ports are excited with equal amplitude and phase difference of $\pi /2$ radians.

### Antenna configuration and simulated performance

**Figure 2:**
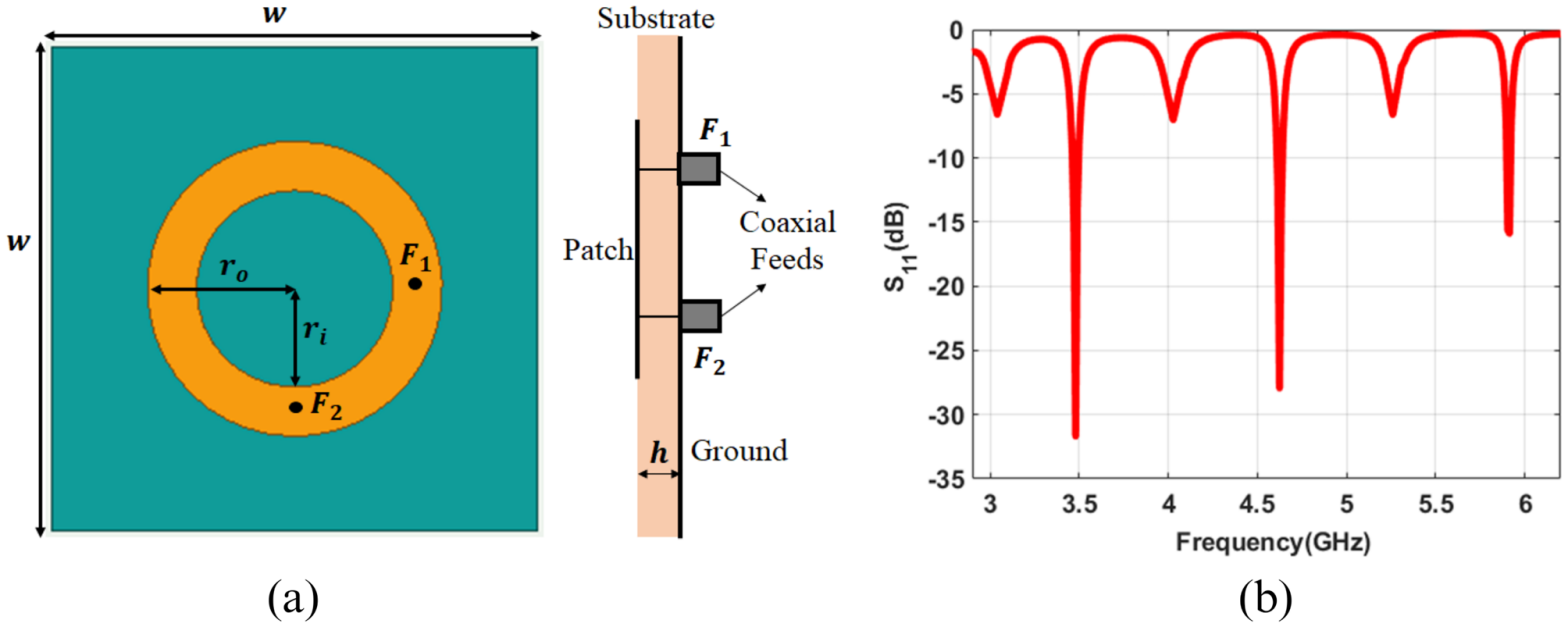
Geometry and simulated reflection coefficient of proposed antenna structure (**a**) Front and side view (**b**) reflection coefficient, where *w*=150 mm, $r_o$=48 mm, $r_i$=32 mm, *h*=2 mm.

Figure 2a represents the geometry of the proposed dual port ring patch antenna to excite circular polarized even order OAM modes $(l = 2, 4~ and~6)$. The proposed ring patch antenna is modeled in Ansys HFSS. The parameters $r_o$ and $r_i$ are the outer and inner radii of the circular ring patch. The antenna is fabricated on a Duroid substrate with a relative permittivity of 2.2, a loss tangent of 0.0009, and a thickness of 1.575 mm. The substrate and ground have the exact dimensions of $l\times w$. The two probe-fed ports have an angular separation of 90 degrees. Both the ports are excited with equal E-field amplitude, which are delayed with $\pi /2$ radians to excite desired circular polarized OAM modes.

The optimized reflection coefficient of the proposed dual port ring patch antenna is shown in Fig. 2b. It can be seen that the proposed ring patch antenna has a return loss value of less than -10 dB in the desired three frequency bands centered at frequencies of 3.49 GHz, 4.63 GHz, and 5.92 GHz corresponding to even order OAM modes $(l = 2, 4~ and~6)$. Three resonance dips with poor impedance matching due to non-circular polarized odd-order oAM modes can also be seen at alternate frequency bands. These odd-order modes are suppressed here by optimizing the inner radius $r_i$ and offset position of both ports from the center of the ring patch. The development steps of the proposed antenna and suppression of undesired odd-order modes are explained in the next subsection.

**Figure 3:**
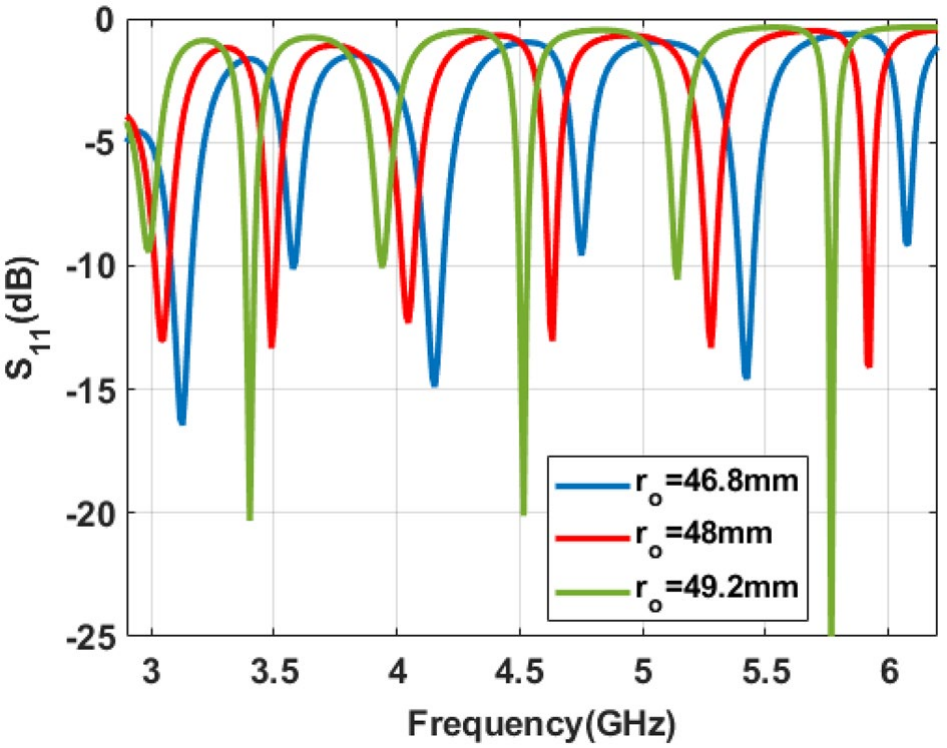
Simulated reflection coefficient of proposed antenna.

### Antenna development steps and suppression of odd order modes

The outer and inner radii, angular separation between both feed points, and offset position of feed points have key roles in exciting desired circular polarized OAM modes and suppressing undesired non-circular polarized OAM modes. These parameters are optimized to excite circular polarization for required even-order OAM modes.

#### Variation in outer radius of the ring patch

When the proposed two-port ring patch antenna is excited with two equal E-field amplitude and $\pi /2$ phase delay, then six resonant modes are observed between 3 and 6 GHz for $r_o=48~mm$ as shown in Fig. 3. It can be seen that the antenna can operate in six frequency bands centered at 3.04GHz, 3.50 GHz, 4.05 GHz, 4.63 GHz, 5.27GHz and 5.92GHz for $r_o=48~mm$. The resonance frequency of these modes can be varied by varying outer radii $r_o$ of the ring patch. It can be observed that the outer effective radius and resonating frequency for a specific mode are inversely related to each other as in Eq. (1).

#### Variation in inner radius and feed offset from center

The impedance matching of specific modes can be improved or reduced by optimizing the inner radii and feed offset of both ports from the center of the ring patch. The goal of this work is to excite only circular polarized OAM modes. So, the odd order OAM modes $(l = 1, 3~ and~5)$ are needed to be suppressed.

**Figure 4:**
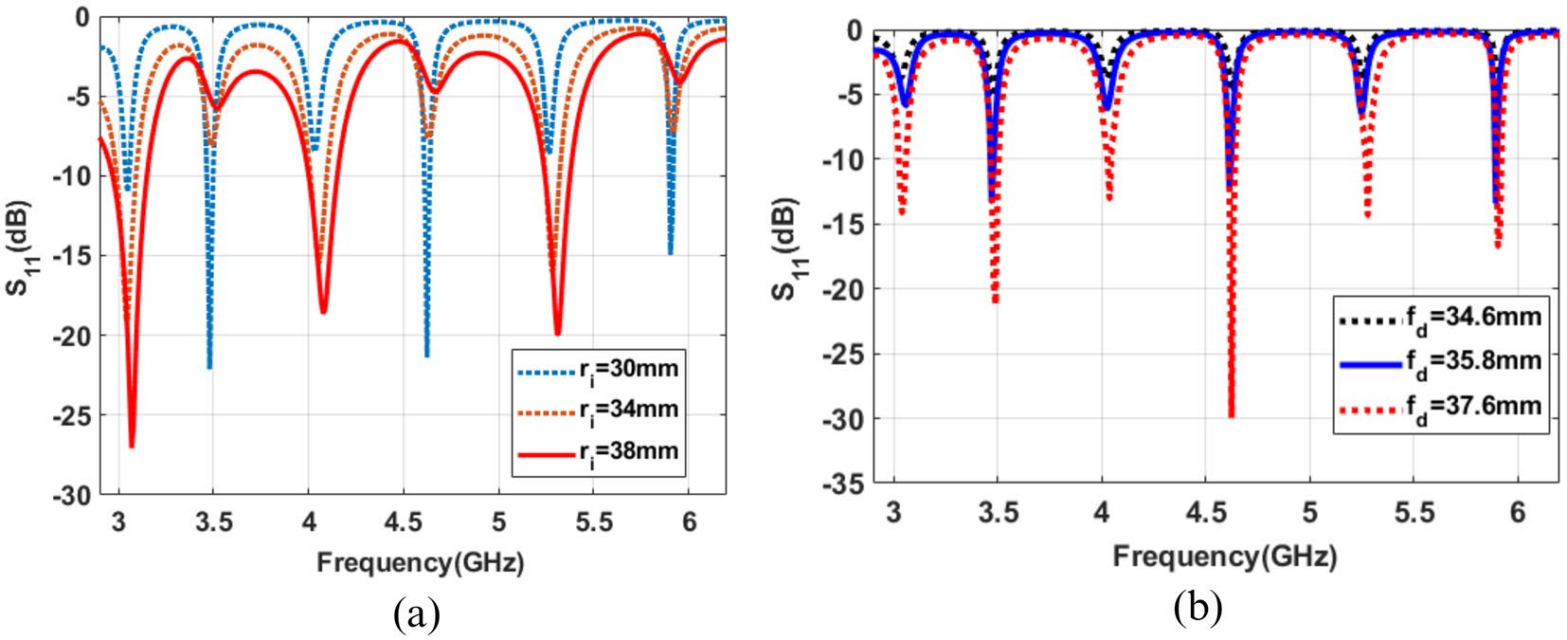
Simulated reflection coefficient (**a**) variation of patch inner radius (**b**) variation of feed distance from the center.

Figure 4a represents the simulated reflection coefficients of the proposed ring patch antenna by varying inner feed radius. It can be observed that the impedance matching of even-order modes can be improved by decreasing the inner radius of the ring patch. However, it reduces impedance matching of odd-order modes. So, odd-order modes can be suppressed by decreasing the inner radius of the ring patch.

The odd order modes can be further suppressed by varying the feed offset $f_d$ of both feeds from the center while keeping the inner and outer radii constant, as shown in Fig. 4. It can be seen that the impedance matching of the odd order OAM modes is significantly reduced at a feed distance of 35.8mm from the center. In contrast, the return loss for all the circular polarized even order modes $(l = 2, 4~ and~6)$ is sufficiently less than −10 dB.

Near-field and far-field characteristics will be analyzed for the proposed ring patch antenna to help identify multiple excited OAM modes.

#### Near field characteristics

**Figure 5:**
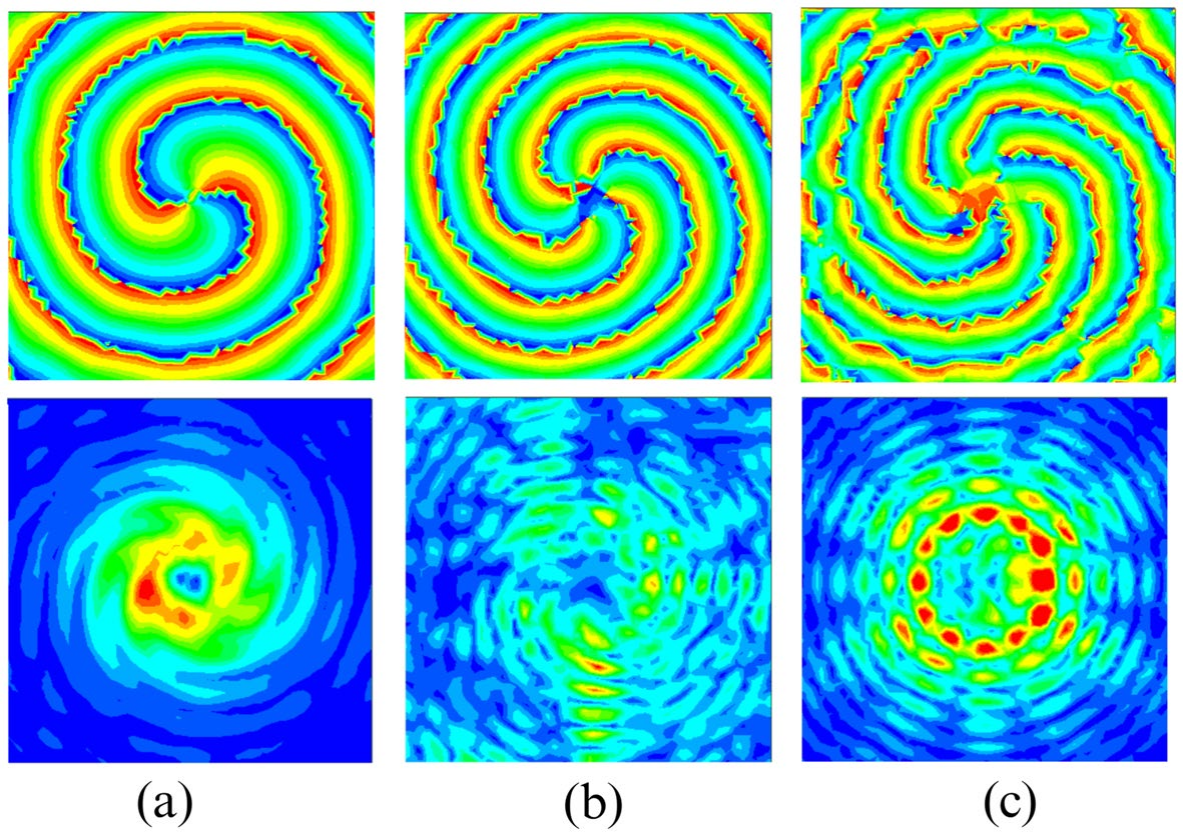
Simulated near field phase and amplitude distribution on the plane at a distance of z=$50\text {mm}$ (**a**) $l=2$ OAM mode ($\mathrm {TM_{31}}$) at 3.49 GHz (**b**) $l=4$ OAM mode ($\mathrm {TM_{51}}$) at 4.63 GHz (**c**) $l=6$ OAM mode ($\mathrm {TM_{71}}$) at 5.92 GHz.

The proposed ring patch antenna’s simulated phase and amplitude of electric field distribution are shown in Fig. 5 at three resonant frequencies of desired even order OAM modes. The field distribution is observed on a rectangular plane of $500\textrm{mm} \times 500\textrm{mm}$ at $\textrm{z}=50\textrm{mm}$. All three distributions have amplitude distribution with null at the center and corresponding spiral phase distribution for even order OAM modes $(l = 2, 4~ and~6)$ at frequencies of 3.49 GHz, 4.63 GHz and 5.92 GHz, respectively, as shown in Fig. 5a,b,c, which confirms the excitation of desired even order OAM modes. It can be seen that the mode order of the generated OAM wave is increased with an increase in resonating frequency.

**Figure 6:**
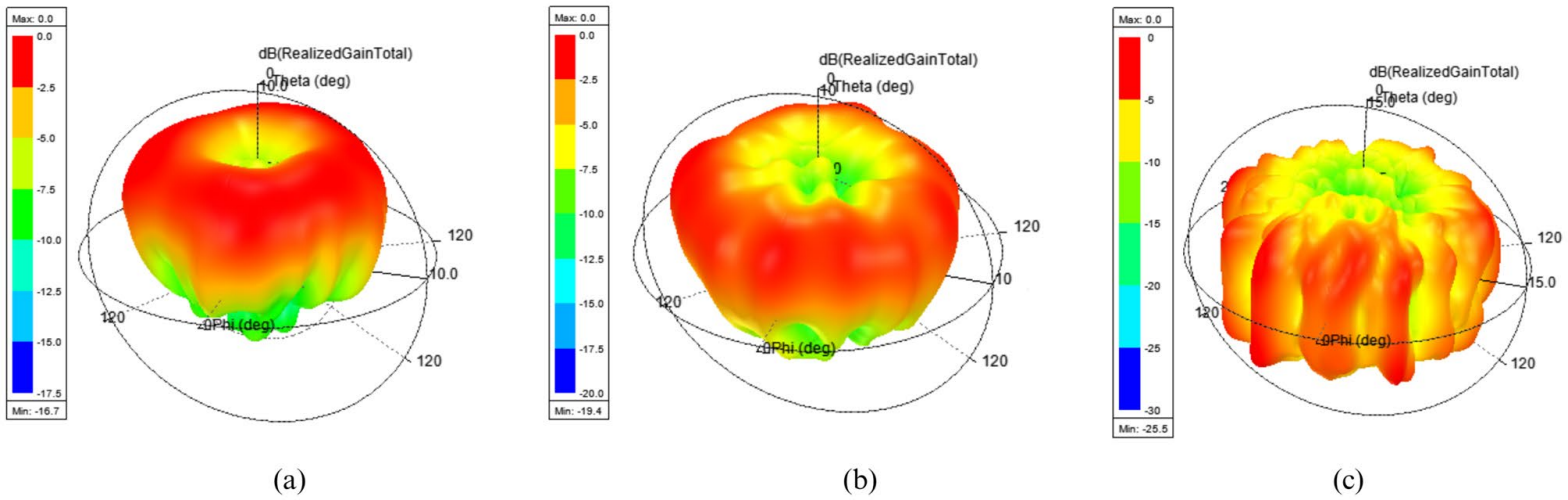
Simulated far field plots of dual port ring patch antenna for even order OAM modes (**a**) mode 2 (**b**) mode 4 (**c**) mode 6.

**Figure 7:**
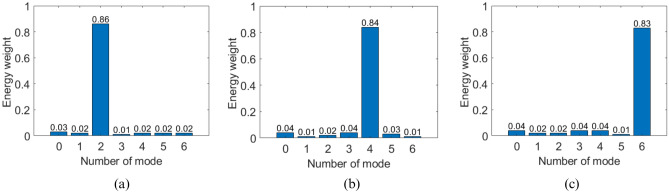
Mode purity of proposed ring patch antenna (**a**) $l=2$ at 3.49 GHz (**b**) $l=4$ at 4.63 GHz (**c**) $l=6$ at 5.92 GHz.

**Figure 8:**
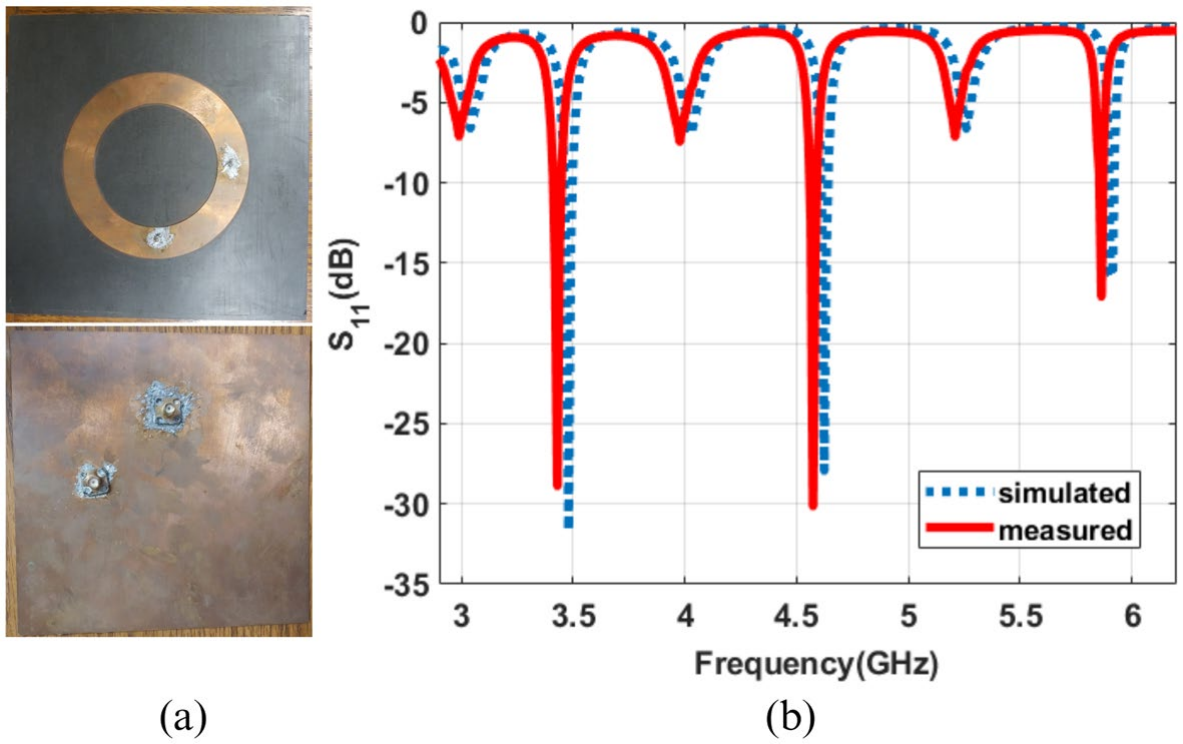
Fabricated prototype (**a**) front and back view (**b**) measured reflection coefficient.

#### Farfield characteristics

Figure 6 represents the normalized simulated far-field pattern of the proposed ring patch antenna for all the desired even-order OAM modes at their corresponding frequencies. It can be seen that all the far-field radiation patterns have desired null along the beam axis, also known as phase vortex, which is the exotic feature of OAM carrying beams^55–57^. The divergence angle of the cone-shaped pattern of OAM carrying beams increases with an increase in OAM mode number, which can be used to cover IoT devices of different geographical regions located at different radial distances as in^42^.

#### OAM mode purity

OAM mode purity is also calculated at the resonance frequency of each frequency band. Figure 7 represents the OAM mode purity of the proposed ring patch antenna for all three frequency bands. It can be seen that the proposed ring patch antenna exhibits good OAM mode purity of 86, 84, and 83 percent for OAM modes $l=2,~4$, and 6, respectively, in corresponding frequency bands. This OAM mode purity analysis confirms the simultaneous excitation of desired multiple OAM modes. It effectively suppresses the undesired modes through a simple configuration of the proposed dual port single-ring patch antenna.

**Figure 9:**
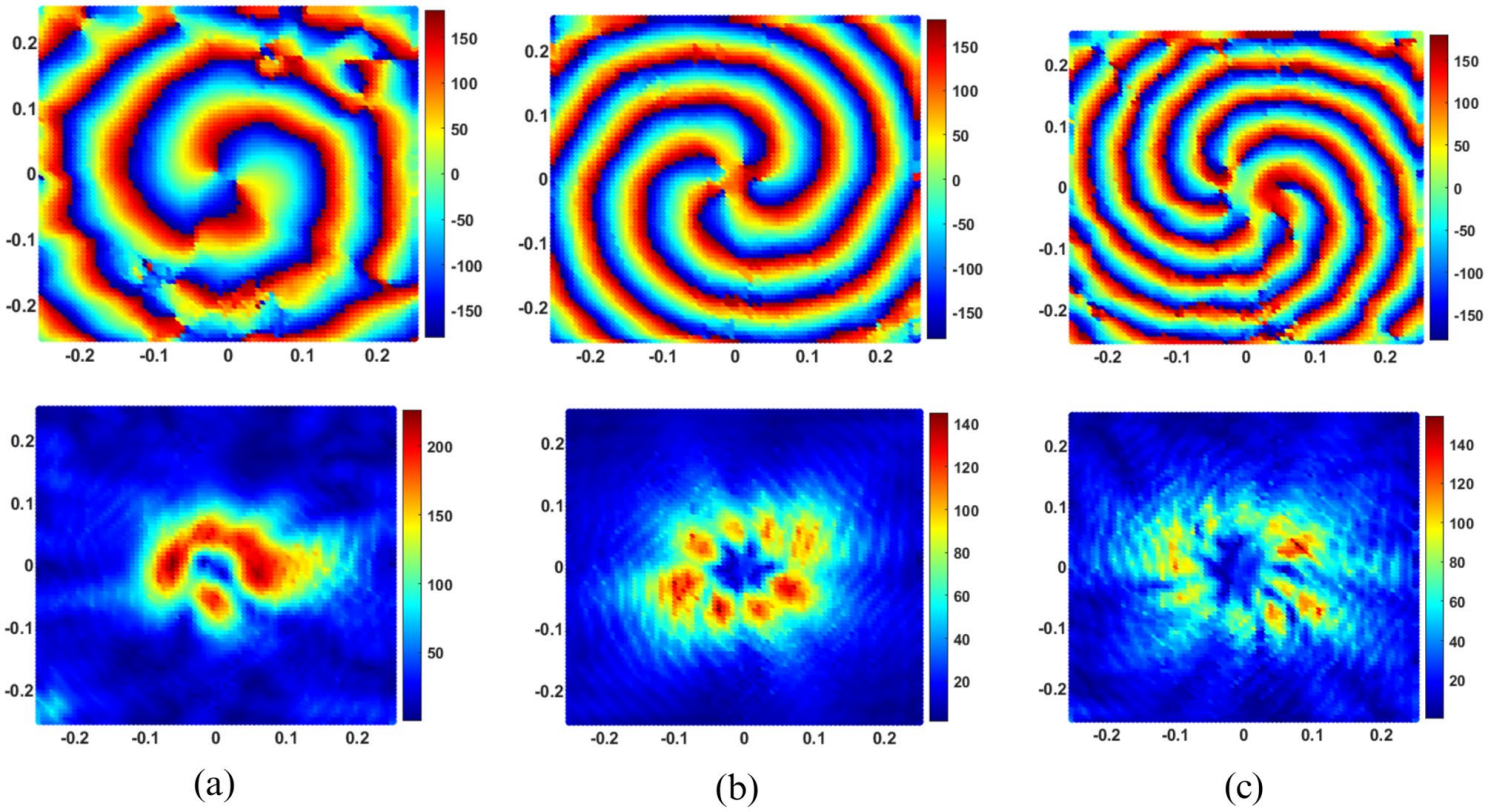
Measured far field results (**a**) $l=2$ OAM mode phase and amplitude at 3.49 GHz (**b**) $l=4$ OAM mode phase and amplitude at 4.63 GHz (**c**) $l=6$ OAM mode phase and amplitude at 5.92 GHz.

## Characterization for fabricated prototype of antenna

### Reflection coefficient

Figure 8a represents the front and back view of the fabricated prototype of the proposed dual port ring patch antenna. Figure 8b shows the proposed antenna’s simulated and measured reflection coefficient. It can be observed that the proposed antenna exhibits good measured impedance characteristics at frequencies of 3.48 GHz, 4.62 GHz, and 5.91 GHz for even-order OAM modes. Small deviations in resonance frequencies of the fabricated prototype are observed due to fabrication tolerance.

### Near field characteristics

The measured near field distribution for phase and amplitude at all three frequencies are shown in Fig. 9a,b,c. Electric field distributions were measured along x and y-directions on a plane of 500 mm×500 mm at a distance of z=50 mm with 7 mm step size in each direction. The measured electric field distributions of phase and amplitude also verify that the proposed dual port ring patch antenna has successfully excited multiband circular polarized multiple OAM modes $(l = 2, 4~ and~6)$.

**Figure 10:**
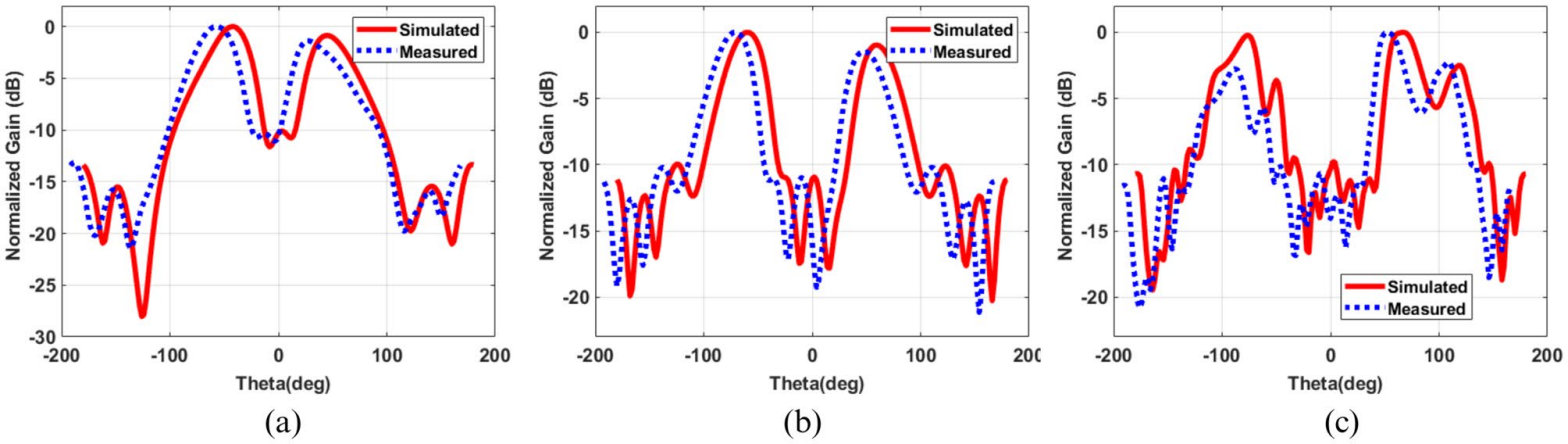
Normalized far field radiation pattern (**a**) $l=2$ at 3.49 GHz (**b**) $l=4$ at 4.63 GHz (**c**) $l=6$ at 5.92 GHz.

**Figure 11:**
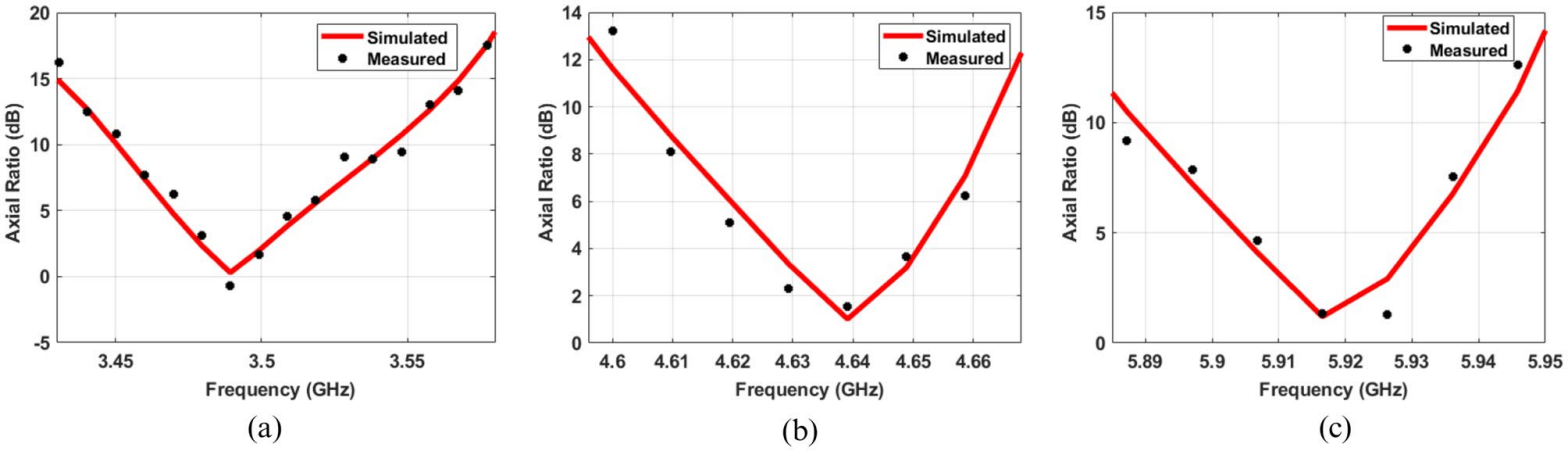
Simulated and measured axial ratio (**a**) $l=2$ at 3.49 GHz (**b**) $l=4$ at 4.63 GHz (**c**) $l=6$ at 5.92 GHz.

### Far field characteristics

The proposed ring patch antenna’s simulated and measured normalized radiation pattern is shown in Fig. 10. It can be observed that the proposed ring patch antenna has significant null in broadside direction at their corresponding frequencies, which also confirms the characteristic of OAM wave. The simulated and measured radiation patterns also have a good match.

### Axial ratio

The simulated and measured axial ratio performance for all three frequency bands of the proposed OAM antenna are shown in Fig. 11. It can be seen that the proposed antenna exhibits less than 3 dB axial ratio in all three frequency bands. The simulated and measured results are also in good agreement. The small fractional bandwidth of the antenna in each frequency band is suitable for narrow band circular polarized IoT applications^58–60^.

The comparison of the proposed antenna with the reported ones is presented in Table 1. It can be observed that the proposed dual port single-ring patch antenna has excited higher-order circular polarized multi-modes at multiple frequencies with less complexity, which is significant in comparison to others.

**Table 1:** Comparison of proposed ring patch antenna with reported ones.

Ref	Frequency (GHz)	Circular polarized (Yes/No)	${TM_{nm}}$ Modes	Complexity
^34^	5.03	Yes	$TM_{41}$	Low
^35^	13.7	Yes	$TM_{31}$	Low
^36^	4.4, 7.8, 9.4	No	$TM_{11}$, $TM_{02}$, $TM_{12}$	High
^37^	5.8	No	$TM_{21}$, $TM_{12}$	High
^38^	12.9	No	$TM_{01}$, $TM_{02}$	High
^39^	18.5	No	$TM_{02}$	High
^40^	10.4	No	$TM_{01}$, $TM_{02}$	High
^41^	5.8	Yes	$TM_{11}$, $TM_{21}$, $TM_{31}$	High
This Work	3.49, 4.63, 5.92	Yes	$TM_{31}$, $TM_{51}$, $TM_{71}$	Low

## Conclusion

This paper proposes a multiple-band multi-mode OAM antenna design using a simple dual-port ring patch, which can excite multiple circular polarized OAM states. Each $TM_{n1}$ mode ($TM_{31}$, $TM_{51}$ and $TM_{71}$) is associated with corresponding OAM states $(l = 2, 4~ and~6)$, respectively, at their corresponding frequencies. Furthermore, the technique is proposed for simultaneous excitation of circularly polarized radiation for even-order OAM modes. In addition, OAM states with opposite signs can also be excited by reversing the phase shift between both feeds. The proposed antenna is suitable for wireless applications in IoT devices.

## Data Availability

The datasets analysed during the current study available from the corresponding author on reasonable request.
