# Author Correction: Realization of circular polarized multiple band multi-mode OAM antenna using a ring patch for IoT applications

**DOI:** 10.1038/s41598-024-51956-1

**Published:** 2024-01-18

**Authors:** Umar Fayyaz, Shahab Ahmad Niazi, Khaled AlJaloud, Abdul Aziz, Waqar Ahmad Malik, Rifaqat Hussain

**Affiliations:** 1 Faculty of Engineering and Technology, The Islamia University of Bahawalpur, Punjab, 63100 Pakistan; 2 College of Engineering, Muzahimiyah Branch, King Saud University, P.O. Box 2454, Riyadh, Saudi Arabia; 3 Department of Avionics Engineering, College of Aeronautical Engineering (CAE), National University of Sciences and Technology (NUST), Risalpur, Khyber‑Pakhtunkhwa Pakistan; 4 Antenna and Electromagnetics Research Group, School of Electronic Engineering and Computer Science, Queen Marry University of London, London, UK

Correction to: *Scientific Reports* 10.1038/s41598-023-43836-x, published online 10 October 2023

The original version of this Article contained an error in the Acknowledgements section.

“This work was supported by Researchers Supporting Project number (RSR2023R474), King Saud University, Riyadh, Saudi Arabia.”

now reads:

“This work was supported by Researchers Supporting Project number (RSP2023R474), King Saud University, Riyadh, Saudi Arabia.”

The original Article has been corrected.

